# Hypoxia preconditioning promotes cardiac stem cell survival and cardiogenic differentiation in vitro involving activation of the HIF-1α/apelin/APJ axis

**DOI:** 10.1186/s13287-017-0673-4

**Published:** 2017-09-29

**Authors:** Jingying Hou, Lei Wang, Huibao Long, Hao Wu, Quanhua Wu, Tingting Zhong, Xuxiang Chen, Changqing Zhou, Tianzhu Guo, Tong Wang

**Affiliations:** 1 Guangdong Provincial Key Laboratory of Malignant Tumor Epigenetics and Gene Regulation, Sun Yat-sen Memorial Hospital of Sun Yat-sen University, 107 Yanjiang Xi Road, Guangzhou, Guangdong 510120 China; 2 Department of Emergency, Sun Yat-sen Memorial Hospital of Sun Yat-sen University, 107 Yanjiang Xi Road, Guangzhou, Guangdong 510120 China; 3 Guangdong Province Key Laboratory of Arrhythmia and Electrophysiology, 107 Yanjiang Xi Road, Guangzhou, Guangdong China

**Keywords:** Hypoxia preconditioning, Cardiac stem cells, HIF-1α, Apelin/APJ, Survival, Cardiogenic differentiation

## Abstract

**Background:**

Cardiac stem cells (CSCs) transplantation has been regarded as an optimal therapeutic approach for cardiovascular disease. However, inferior survival and low differentiation efficiency of these cells in the local infarct site reduce their therapeutic efficacy. In this study, we investigated the influence of hypoxia preconditioning (HP) on CSCs survival and cardiogenic differentiation in vitro and explored the relevant mechanism.

**Methods:**

CSCs were obtained from Sprague–Dawley rats and cells of the third passage were cultured in vitro and exposed to hypoxia (1% O_2_). Cells survival and apoptosis were evaluated by MTS assay and flow cytometry respectively. Cardiogenic differentiation was induced by using 5-azacytidine for another 24 h after the cells experienced HP. Normoxia (20% O_2_) was used as a negative control during the whole process. Cardiogenic differentiation was assessed 2 weeks after the induction. Relevant molecules were examined after HP and during the differentiation process. Anti-hypoxia-inducible factor-1α (HIF-1α) small interfering RNA (siRNA), anti-apelin siRNA, and anti-putative receptor protein related to the angiotensin receptor AT1 (APJ) siRNA were transfected in order to block their expression, and relevant downstream molecules were detected.

**Results:**

Compared with the normoxia group, the hypoxia group presented more rapid growth at time points of 12 and 24 h (*p* < 0.01). Cells exhibited the highest proliferation rate at the time point of 24 h (*p* < 0.01). The cell apoptosis rate significantly declined after 24 h of hypoxia exposure (*p* < 0.01). Expression levels of HIF-1α, apelin, and APJ were all enhanced after HP. The percentage of apelin, α-SA, and cTnT positive cells was greatly increased in the HP group after 2 weeks of induction. The protein level of α-SA and cTnT was also significantly elevated at 7 and 14 days (*p* < 0.01). HIF-1α, apelin, and APJ were all increased at different time points during the cardiogenic differentiation process (*p* < 0.01). Knockdown of HIF-1α, apelin or APJ by siRNAs resulted in a significant reduction of α-SA and cTnT. HIF-1α blockage caused a remarkable decrease of apelin and APJ (*p* < 0.01). Expression levels of apelin and APJ were depressed after the inhibition of apelin (*p* < 0.01).

**Conclusion:**

HP could effectively promote CSCs survival and cardiogenic differentiation in vitro, and this procedure involved activation of the HIF-1α/apelin/APJ axis. This study provided a new perspective for exploring novel strategies to enhance CSCs transplantation efficiency.

## Background

Cardiac stem cell (CSC) transplantation represents an optimal therapeutic approach for cardiac regeneration after myocardial infarction (MI). CSCs are lineage specific to the cardiac phenotype and have been considered a logical candidate for reconstituting the lost myocardium [1, 2]. Various preclinical and clinical studies have demonstrated the reduction of infarct size, amelioration of ventricular remodeling, enhancement of heart function, and improvement of electrophysiological stability post transplantation of these cells [2–4]. CSCs generate functionally competent myocardium and form stable electromechanical couplings with the host cardiomyocytes, which significantly reduces the occurrence of malignant arrhythmia [3–5]. However, inferior survival and low differentiation efficiency of these cells restrict their further clinical application [6]. It is imperative to search for new strategies to promote their survival and cardiac differentiation.

Accumulating evidence reveals that hypoxia influences stem cell survival and cardiomyocyte differentiation [7]. A lower oxygen level improves viability of mesenchymal stem cells (MSCs) and cardiac progenitor cells (CPCs) [8, 9]. In addition, hypoxia facilitates cardiomyocyte differentiation of embryonic stem cells (ESCs) and MSCs [10, 11]. Hypoxia stress initiates an alteration of cardiomyocyte differentiation-related genes and proteins [11]. Hypoxia-inducible factor-1α (HIF-1α) is an oxygen-sensitive transcription factor and dominates most of the biological effects mediated by hypoxia [12]. It has been revealed that upregulating HIF-1α by hypoxia preconditioning (HP) can remarkably strengthen the proliferation and survival of MSCs and CPCs and augment their therapeutic potential [13–15]. Other studies show that HIF-1α propels cardiomyogenesis of ESCs and MSCs [10, 11]. However, the direct impact of hypoxia on CSCs cardiogenic differentiation and the regulatory role of HIF-1α in the process remain to be elucidated.

Apelin, a newly discovered peptide, is the endogenous ligand for putative receptor protein related to the angiotensin receptor AT1 (APJ). Apelin/APJ performs a crucial role in the maintenance of heart development and cardiovascular homeostasis [16]. Recent studies have confirmed that apelin/APJ is tightly linked with stem cell survival and cardiomyocyte differentiation [17–19]. Previous data indicate that apelin/APJ is a vital downstream signal of HIF-1α under the hypoxic condition [20]. HIF-1α binds to the endogenous hypoxia-responsive element (HRE) site of the apelin gene to induce its expression [21]. In this study, we investigated the influence of HP on CSCs survival and cardiogenic differentiation in vitro and explored the role of the HIF-1α/apelin/APJ axis in the procedure.

## Methods

### Ethics statement

Ten Sprague–Dawley (SD) rats weighing 25 g were obtained from the Animal Experimental Center of the Sun Yat-sen University. All animal handling and procedures were performed in accordance with protocols approved by the Animal Ethics Committee of Sun Yat-sen University (201611024).

### Isolation and culture of CSCs

Under sterile conditions, the heart of a 3-day-old newborn Sprague–Dawley male rat was excised, put into the aseptic culture dish, and minced into 1-mm^3^ pieces. The pieces were then washed twice with phosphate buffer solution (PBS) in order to remove impurities. Then 2–3 ml of 0.2% trypsin were added for 5 min of digestion, followed by 2–3 ml of 0.1% collagenase II for 5 min for additional digestion, and subsequent elimination of the digestive liquid. The procedure was then repeated an additional three times. The pieces were then washed twice with complete explant medium (CEM; supplemented with Iscove’s Modified Dulbecco’s Medium, 10% fetal calf serum, 100 U/ml penicillin G, 100 μg/ml streptomycin, 2 mmol/l l-glutamine, 0.1 mmol/l 2-mercaptoethanol) and then transferred and dispatched with a pipette into a 25-cm^2^ flask and incubated with 1 ml CEM at 37 °C in a humidified atmosphere with 5% CO_2_ for 12 h. An additional CEM (3–4 ml) was then added. At 90% confluence, the cells were trypsinized (0.25% trypsin–EDTA, Catalog No. 25-053-CI; Mediatech, Hendon, VA, USA) and were passed into 25-cm^2^ flasks at 1:2 ratios. Third-passage CSCs were used in the experiment. Cultured CSCs were analyzed by fluorescence-activated cell sorting (FACScan flow cytometer; Becton Dickinson, Sparks, MD, USA) as reported previously [3, 4, 22], Cell markers used in FACS were c-kit, CD29, CD90.1, CD11b/c, CD34, and CD45. The third-passage CSCs were positive for c-kit, CD29, and CD90.1 and negative for CD11b/c, CD34, and CD45 [3, 4, 22].

### Hypoxia treatment of CSCs

Cells in the hypoxia group were incubated in complete media in 1% O_2_ in a Galaxy® 48 R incubator (Eppendorf/Galaxy Corporation, USA) at 37 °C. Normoxia (20% O_2_) was used as a negative control during the whole experiments.

### Cells proliferation assay

Cells of different groups were collected and suspended in complete culture medium (Hyclone, USA). The growth curve and the 3-(4,5-dimethylthiazol-2-yl)-5-(3-carboxymethoxyphenyl)-2-(4-sulfophenyl)-2H-tetrazolium (MTS) cell viability assay (cellTiter96AQ, one solution cell proliferation assay, Catalog No. G3582; Promega, USA) were adopted to evaluate survival and proliferation ability of MSCs. A total of 1 × 10^5^ cells were seeded equally into each well on 96-well plates. MTS was added to the medium at a final concentration of 0.5 mg/ml for 4 h. The optimal density values (OD490) were read from cellTiter96AQ at different time points respectively. The proliferation rate was calculated as described previously [23, 24]:

Proliferation rate = [OD values at other time points/OD value at the beginning (for the same sample)] × 100%.

### Apoptosis analysis by flow cytometry

The percentage of apoptosis cells was detected by flow cytometry. Cells were washed twice with PBS and 1 × 10^6^ cells were resuspended in Annexin-V binding buffer. Cells were mixed with 5 μl Annexin V-FITC and incubated at room temperature for 15 min. Then 20 μg/ml PI was added afterward. Cells were washed twice and resuspended in 400 μl of annexin-binding buffer. Analysis was performed on flow cytometry.

### Cardiogenic differentiation of CSCs

Differentiation of CSCs into cardiogenic cells was accomplished after 24-h exposure to hypoxia or normoxia. Cells of the two groups were seeded into six-well plates at a concentration of 1 × 10^6^ cells per well. To induce cell differentiation, the cells were incubated in a medium containing 5-azacytidine (5-AZA, 10 μM; Sigma–Aldrich) for 24 h at 37 °C in a humidified atmosphere with 5% CO_2_. The cells were then washed twice and the medium was replaced with normal DMEM. The medium was changed every 3 days and this procedure was terminated at 2 weeks. The morphological changes in CSCs were observed under a microscope (CX41; Olympus).

### Immunofluorescence staining

Slides with the treated cell samples taken from the dishes were used directly. After drying at room temperature for a few minutes, they were permeabilized in 2% formaldehyde/PBS for 10 min. Antigen retrieval was followed by microwaving sections in sodium citrate buffer (1 M, pH 6.1). Sections were blocked by 5% bovine serum albumin (BSA) at room temperature before incubating with primary antibodies at 4 °C overnight (dilution: apelin, 1:100; cTnT, 1:100; α-SA, 1:500). After washing, sections were incubated with appropriate secondary antibodies and slides were counterstained with 4-6-diamidino-2-phenylindole (DAPI). Images were taken by fluorescent microscopy (Leica, German) with a CCD camera (Tokyo, Japan).

### siRNA experiments

CSCs were incubated at 1 × 10^6^ cells per well in six-well plates at day 0 with siRNAs against HIF-1α (NM_024359; Sigma), apelin (NM_031612; Sigma), and APJ (NM_031349; Sigma). The control siRNAs were used as negative control (NC; Sigma). Transfection of siRNAs was performed using Lipofectamine 2000 (Invitrogen) according to the manufacturer's instructions. HIF-1α, apelin, and APJ knockdown was determined by quantitative real-time PCR.

### Western blot analysis

Protein levels were measured by western blot analysis. Cells were washed several times with PBS before collection and lysed with modified RIPA buffer. Cells were completely lysed after repeated vortexing and supernatants were acquired though centrifugation at 14,000 × *g* for 20 min. Proteins were resolved by sodium dodecyl sulfate–polyacrylamide gel (SDS-PAGE) and afterward transferred to a polyvinylidenedifluoride (PVDF) membrane (IPVH00010; Millipore, Boston, MA, USA) before incubating with primary antibodies (α-SA, Catalog No. ab72592, UK; cTnT, Catalog No. ab45932, UK; HIF-1α, Catalog No. GTX 127309, USA; apelin, Catalog No. ab125213, UK; APJ, Catalog No. LS-C149246, USA). The membranes were subjected to three 5-min washes with TBST and incubated with anti-IgG horseradish peroxidase-conjugated secondary antibody (Southern Biotech, Birmingham, AL, USA) for 60 min at room temperature. After extensive washing, bands were detected by enhanced chemiluminescence. The band intensities were quantified using image software (image J 2×, version 2.1.4.7).

### Quantitative real-time PCR

Total RNA was isolated from cells using a Trizol reagent (Invitrogen) followed by digestion with RNase-free DNase (Promega). Concentration and integrity of total RNA were estimated and the real-time polymerase chain reaction (RT-PCR) was conducted on an ABI PRISM® 7500 Sequence Detection System using SYBR Green qPCR SuperMix (Invitrogen). The primers included rat apelin primer against NM_031612.2 (Catalog No. RQP051208; GeneCopoeia, USA), rat HIF-1α primer against NM_024359.1 (Catalog No. RQP050798; GeneCopoeia, USA), and rat APJ primer against NM_031349.2 (Catalog No. RQP051101; GeneCopoeia, USA). Specific products were amplified and detected with Applied Biosystems at 95 °C for 10 min, followed by 40 cycles at 95 °C for 15 s and at 60 °C for 30 s, at which point data were acquired. The relative level of mRNA was calculated using the 2^−ΔΔCt^ method. For the assays of the molecules examined, the results were quantified as the threshold cycle of each target gene and normalized into the ΔCt value. Quantifications of fold-change in gene expressions were also performed using the 2^−ΔΔCt^ method.

### Statistical analysis

All quantitative data were described as mean ± SD. Statistical analysis was performed using SPSS 16.0 software for Windows. Data were recorded as mean ± SD. The Student’s *t* test was used for comparisons between two groups. *p* <0.05 was considered statistically significant.

## Results

### Hypoxia exposure affected the proliferation of CSCs

**Fig. 1:**
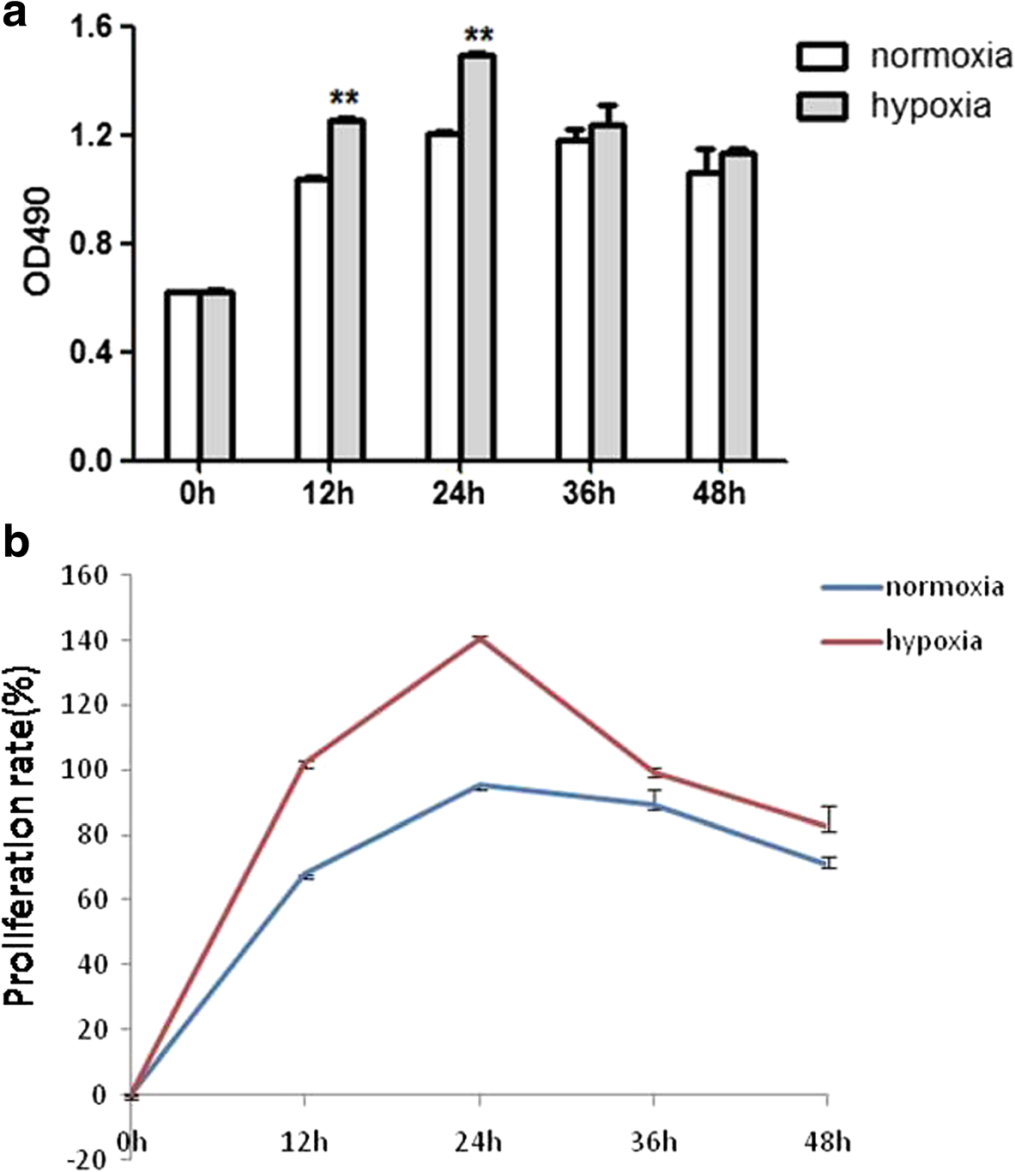
CSCs proliferation at different time points after hypoxia exposure. **a** CSCs proliferation rate tested by MTS. **b** Detection of the proliferation rate at different time points. Proliferation rate = OD (optical density) values at other time points divided by OD value at the beginning (same sample) × 100%. **p < 0.01 vs normoxia

The MTS assay was performed to detect whether hypoxia could affect CSCs proliferation. The hypoxia group displayed a more rapid growth at the time points of 12 and 24 h in contrast with the normoxia group (*p* < 0.01; Fig. 1). Cells presented the highest proliferation rate at the time point of 24 h (*p* < 0.01; Fig. 1b), indicating that 24-h hypoxia exposure generated the greatest facilitative effect on CSCs proliferation. However, cell proliferation showed no difference between the hypoxia and normoxia groups at the time points of 36 and 48 h (*p* > 0.05; Fig. 1). These results implied that 24 h hypoxia pretreatment could efficiently promote the proliferation of CSCs.

### Hypoxia exposure for 24 h reduced the apoptosis of CSCs

**Fig. 2:**
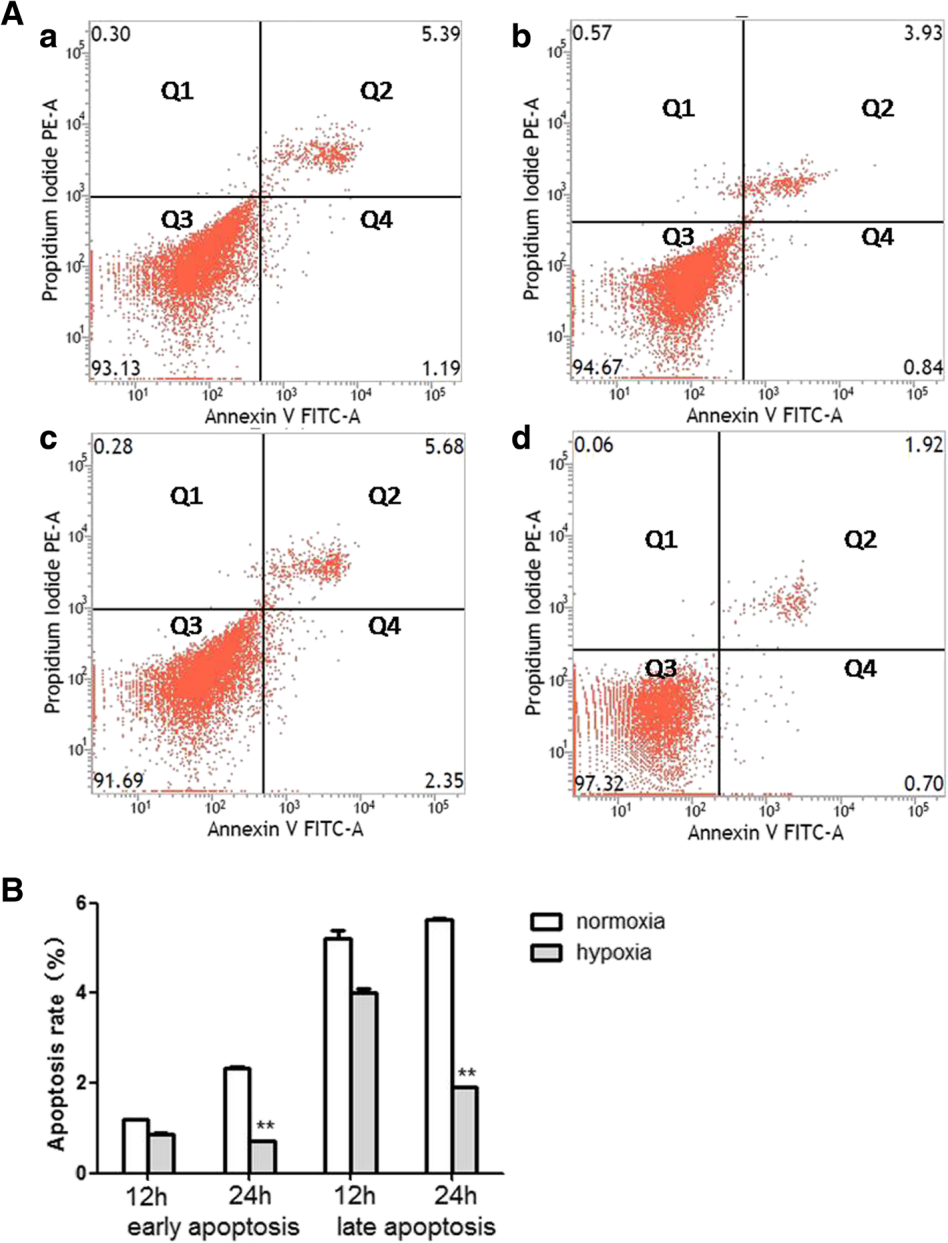
Hypoxia exposure for 24 h reduced the apoptosis of CSCs. (**A**) Apoptosis of CSCs was evaluated by flow cytometry: (a, b) apoptosis rate of CSCs that experienced 12 h normoxia or hypoxia exposure respectively; (c, d) apoptosis rate of CSCs that experienced 24 - normoxia or hypoxia exposure respectively. Quadrant cells were divided into four sections: Q1, Annexin V^−^FITC^−^PI^+^, mechanical error; Q2, Annexin V^−^FITC^+^PI^+^, late apoptosis or necrosis cells; Q3, Annexin V^−^FITC^−^PI^−^, viable cells; Q4, Annexin V^−^FITC^+^PI^−^, early apoptosis cells. (**B**) Comparison of apoptosis between normoxia and hypoxia groups at 12 and 24 h. *n* = 3, **p < 0.01 vs normoxia. FITC fluorescein isothiocyanate, PE phycoerythrin

Cells were stained with Annexin V-FITC/PI, and the effect of hypoxia on cell apoptosis was analyzed by flow cytometer (*p* < 0.01; Fig. 2A). It was indicated that hypoxia exposure for 24 h significantly reduced the proportion of early apoptosis and late apoptosis (*p* < 0.01; Fig. 2B). Nevertheless, hypoxia exposure for 12 h did not cause any significant change of the apoptosis rate (*p* > 0.05; Fig. 2B). This result showed that 24 h hypoxia exposure could attenuate the apoptosis of CSCs.

### Alterations of molecules in the HIF-1α/apelin/APJ axis after HP

**Fig. 3:**
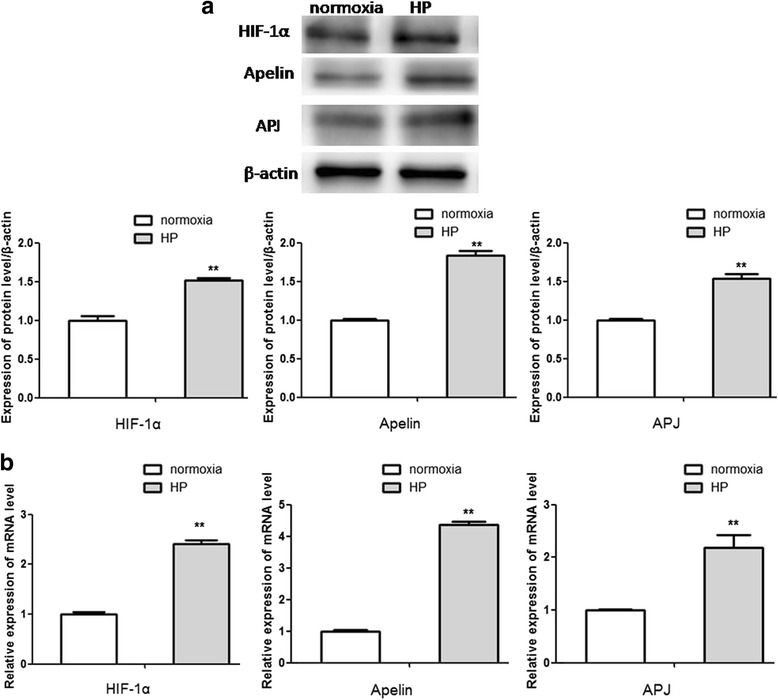
Alterations of molecules in the HIF-1α/apelin/APJ axis after HP. Western blot analysis (**a**) and qRT-PCR (**b**) were used to detect alterations of molecules in the HIF-1α/apelin/APJ axis after HP. Comparisons were made between normoxia and HP groups. **p < 0.01 vs normoxia. HP: hypoxia preconditioning, HIF: hypoxia-inducible factor, apelin: ligand for putative receptor protein related to angiotensin receptor AT1, APJ: putative receptor protein related to angiotensin receptor AT1

Because 24 h hypoxia exposure could dramatically boost CSCs proliferation and repress their apoptosis, cells experiencing hypoxia exposure for 24 h were defined as the HP group in subsequent experiments. In order to investigate the role of the HIF-1α/apelin/APJ axis in hypoxia-mediated CSCs survival, alterations of molecules in the HIF-1α/apelin/APJ axis were explored after HP. The protein and mRNA levels of HIF-1α, apelin, and APJ were all significantly increased in the HP group in contrast with the normoxia group (Fig. 3a, b), implying that the HIF-1α/apelin/APJ axis was activated after HP.

### Cardiogenic differentiation of CSCs after HP

**Fig. 4:**
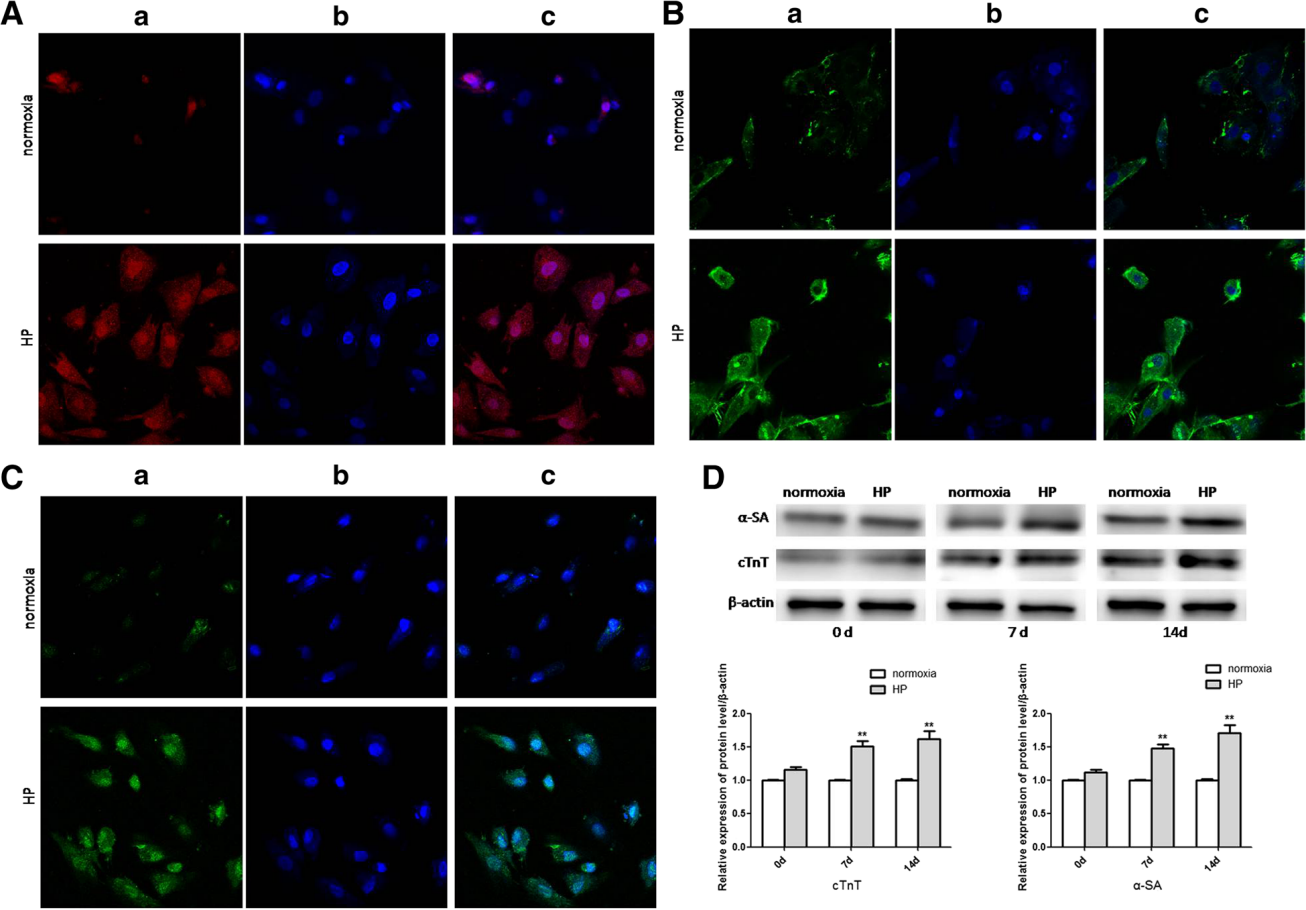
Cardiogenic differentiation of CSCs. (**A**), (**B**), (**C**) Confocal microscopy of immunofluorescent staining of DAPI-labeled CSCs induced by 5-AZA after 14 days (400×). (**A**) Immunofluorescent staining of apelin: (a) cells stained with antibody to apelin appeared red; (b) cells derived from DAPI-labeled CSCs induced by 5-AZA displayed blue nuclei; (c) merged image of a and b. (**B**), (**C**) Immunofluorescent staining of α-SA and cTnT respectively: (a) cells stained with antibody to α-SA and cTnT appeared green; (b) cells derived from DAPI-labeled CSCs induced by 5-AZA displayed blue nuclei; (c) merged image of a and b. (**D**) Alterations of the protein expressions of cardiogenic differentiation specific genes. Western blot analysis to detect alterations of protein expressions of cardiogenic differentiation specific genes including α-SA and cTnT at different time points after induction of cardiogenic differentiation. Comparisons were made between normoxia and HP groups. **p < 0.01 vs normoxia. HP: hypoxia preconditioning

Immunofluorescence staining was applied to detect the effect of HP on cardiogenic differentiation of CSCs. There was an obviously higher percentage of apelin (red, Fig. 4A), α-SA (green, Fig. 4B), and cTnT (green, Fig. 4C) positive cells in the HP group after 2 weeks of induction. The differentiated CSCs expressed cardiac specific markers including α-SA and cTnT. The protein levels of α-SA and cTnT were elevated prominently at the time points of 7 and 14 days after the induction of cardiogenic differentiation (Fig. 4D). These results indicated that HP could impel cardiogenic differentiation of CSCs.

### Alterations of molecules in the HIF-1α/apelin/APJ axis during cardiogenic differentiation of CSCs

**Fig. 5:**
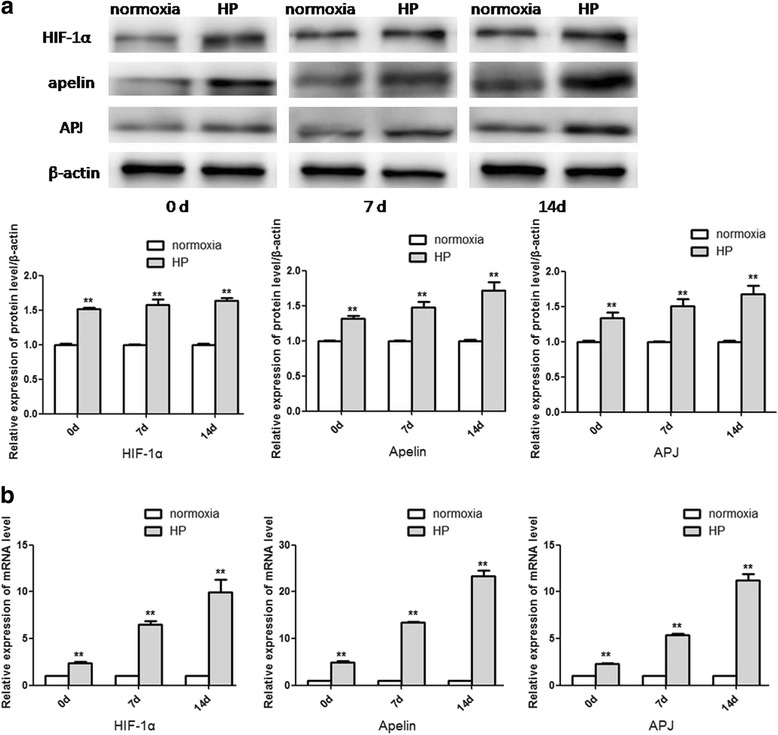
Alterations of molecules in the HIF-1α/apelin/APJ axis after the induction of cardiogenic differentiation. Western blot analysis (**a**) and qRT-PCR (**b**) were used to detect the expressions of molecules in the HIF-1α/apelin/APJ axis at different time points after the induction of cardiogenic differentiation. Comparisons were made between normoxia and HP groups. **p < 0.01 vs normoxia. HP hypoxia preconditioning, HIF: hypoxia-inducible factor, apelin: ligand for putative receptor protein related to angiotensin receptor AT1, APJ: putative receptor protein related to angiotensin receptor AT1

In order to identify the function of the HIF-1α/apelin/APJ axis in HP-mediated cardiogenic differentiation of CSCs, alterations of molecules in the HIF-1α/apelin/APJ axis were explored in different groups during cardiogenic differentiation. The protein and mRNA levels of HIF-1α, apelin, and APJ were all significantly increased at different time points in the HP group in contrast with the normoxia group after the induction (Fig. 5a, b), suggesting that the HIF-1α/apelin/APJ axis was persistently activated during cardiogenic differentiation of CSCs after HP.

### Inhibition of the HIF-1α/apelin/APJ axis interfered with HP-mediated cardiogenic differentiation of CSCs

**Fig. 6:**
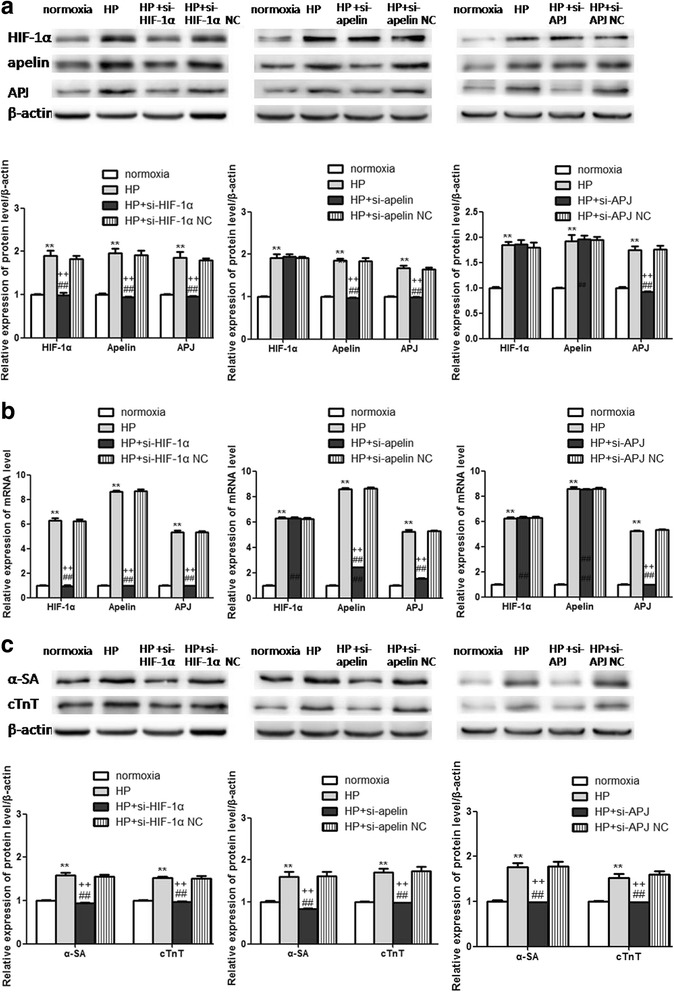
Alterations of molecules and specific markers of cardiogenic differentiation after inhibition of the HIF-1α/apelin/APJ axis. (**a**) Western blot and (**b**) qRT-PCR analysis of the molecules in the HIF-1α/apelin/APJ axis after relevant siRNA interference. **c** Changes of cardiogenic differentiation specific genes including α-SA and cTnT after the blockage of the HIF-1α/apelin/APJ axis. **p < 0.01 vs normoxia, ## *p* < 0.01 vs HP, ††*p* < 0.01 vs NC. HP: hypoxia preconditioning, HIF: hypoxia-inducible factor, apelin: ligand for putative receptor protein related to angiotensin receptor AT1, APJ: putative receptor protein related to angiotensin receptor AT1, NC negative control

Molecules in the HIF-1α/apelin/APJ axis were blocked in order to validate their regulatory role in HP-mediated effects. Anti-HIF-1α siRNA (si-HIF-1α,), anti-apelin siRNA (si-apelin), and anti-APJ siRNA (si-APJ) and their control siRNAs (NC) were transiently transfected into undifferentiated CSCs before HP and further induction of cardiogenic differentiation. Expression levels of relevant molecules were analyzed 72 h later. In contrast with the normoxia group, there was an obvious upregulation of HIF-1α, apelin, and APJ in the HP group, and cardiac differentiation specific markers including α-SA and cTnT were also increased. Compared with the HP and NC groups, the si-HIF-1α group exhibited a significant reduction of HIF-1α, apelin, and APJ (*p* < 0.01; Fig. 6a, b). The si-apelin group showed a distinct downregulation of both apelin and APJ (*p* < 0.01; Fig. 6a, b). The expression of APJ in the si-APJ group was decreased obviously (*p* < 0.01; Fig. 6a, b). However, there was no alteration of HIF-1α in the si-apelin and si-APJ groups, and no change of the expression of apelin was observed in the si-APJ group. Protein levels of α-SA and cTnT were remarkably reduced in the three siRNA groups (*p* < 0.01; Fig. 6c). The aforementioned results supported that HP-mediated CSCs survival and cardiogenic differentiation could be partially ascribed to activation of the HIF-1α/apelin/APJ axis.

## Discussion

The present study demonstrated that HP promoted CSCs survival and cardiogenic differentiation in vitro, and this procedure involved activation of the HIF-1α/apelin/APJ axis.

CSCs have been studied with great interest due to their natural location and function in the heart. Previous data have verified their beneficial effects in the improvement of cardiac function after transplantation [25]. CSCs are intrinsically programmed to differentiate toward myocardial lineages [1]. As natural resident cells, they could be the ideal candidate for the restoration of the injured cardiomyocytes after infarction. Our previous work has also demonstrated that CSCs can differentiate into cardiomyocytes and express connexin-43 (Cx43) in the infarct zone and border zone after transplantation [3, 4]. However, the specific mechanism that mediates the CSCs cardiogenic differentiation in the hypoxic-ischemic microenvironment in the local infarct site is still enigmatic.

Oxygen tension is a mediator of stem cell plasticity, proliferation, and differentiation [26]. The potential role of hypoxia signaling in the regulation of CPCs has already been reported. Hypoxia signaling is a vital hallmark of cycling cardiomyocytes [27]. In this study, we tried to investigate the effect of hypoxia on CSCs survival and differentiation. It was shown that the CSCs proliferation rate was increased at the time points of 12 and 24 h in the hypoxia group. The hypoxia group presented the highest proliferation at the time point of 24 h, and cell apoptosis was also significantly decreased after 24 - hypoxia exposure. These results suggested that hypoxia exposure for 24 h could effectively promote CSCs survival and reduce their apoptosis. In view of this, CSCs pretreated in the hypoxic environment for 24 h were defined as the HP group in subsequent experiments. Cardiogenic differentiation of CSCs was detected afterward. We discovered that there was a much higher proportion of cells with the cardiogenic phenotype in the HP group after 2 weeks of the induction. Specific markers of cardiogenic differentiation including α-SA and cTnT were also distinctly increased in the HP group at 7 and 14 d. These results revealed that HP could drive cardiogenic differentiation of CSCs in vitro.

As HP facilitated CSCs survival and cardiogenic differentiation, the underlying mechanism was explored. HIF-1α is a pivotal modulator of oxygen homeostasis and predominantly mediates multiple adaptive responses to hypoxia [12]. Stabilization of the HIF-1α subunit is crucial for the maintenance and function of stem or progenitor cells that reside in relatively hypoxic microenvironments [28]. Evidence exhibits that HIF-1α exerts a positive regulatory role in stem cell survival and cardiac differentiation. Overexpression of HIF-1α contributes to increased tolerance of CPCs under hypoxic stress [29]. HIF-1α upregulation evokes the mobilization of endogenous c-kit (+) CPCs and myocardial angiogenesis, resulting in the improvement of cardiac function [30]. In this study, HP induced a significant upregulation of HIF-1α, which exerted protective effects on CSCs survival in the hypoxic environment. Series studies have confirmed the beneficial role of HIF-1α in mediating cardiac differentiation of stem cells. Exogenous transduction of HIF-1α significantly boosts cardiogenesis of ESCs [31], whereas destabilization of HIF-1α or its knockout blocks the early cardiac differentiation of ESCs under hypoxia [10]. It has been shown that stabilized expression of HIF-1α triggers MSCs differentiation to cardiomyogenic cells under normoxic conditions [32]. Moreover, HIF-1α can assist transdifferentiation of neonatal cardiac fibroblasts into the cardiomyocyte phenotype [33]. In this study, HP propelled cardiogenic differentiation of CSCs. The HP group retained a consistently increased level of HIF-1α during the whole cardiogenic differentiation process. Specific markets of cardiac differentiation including α-SA and cTnT were distinctly elevated. Inhibition of HIF-1α led to a significant downregulation of α-SA and cTnT in the condition of HP. All of these results manifested that HP-mediated CSCs cardiogenic differentiation might be associated with the continuous upregulation of HIF-1α.

Current studies indicate that apelin/APJ vividly participates in stem cell survival and cardiac differentiation. Apelin/APJ promotes survival of MSCs and reduces their apoptosis [17, 18]. One latest study shows that apelin enhances mobilization, survival, and proliferation of endogenous CSCs in the injured heart for cardiac repair post MI [34]. In this study, it was also found that the expressions of apelin and APJ were significantly intensified after HP, indicating that activation of apelin/APJ might perform a critical role in promoting CSCs survival after hypoxia exposure. Apelin/APJ has drawn much attention recently due to the capability of inducing cardiomyocyte differentiation of stem cells. Exogenous apelin can initiate cardiac differentiation of mouse and human ESCs [10, 31, 35]. Sustained expression of apelin/APJ has been discovered during MSCs differentiation into cardiomyogenic cells, and this may be an important mechanism of myocardial repair after MSCs transplantation [36]. Further research indicates that apelin upregulates cardiac specific genes and guides cardiac differentiation of MSCs [37]. In this study, we investigated the alterations of apelin/APJ during cardiogenic differentiation of CSCs. The percentage of apelin positive cells was much higher after HP. There was a continuous increase of apelin/APJ during the whole cardiogenic differentiation procedure. Both apelin and APJ knockdown caused a significant decrease of cardiac differentiation markers including α-SA and cTnT in the condition of HP. The aforementioned results showed that apelin/APJ might be indispensible for HP-induced CSCs cardiogenic differentiation in vitro.

Apelin/APJ has been identified as a vital downstream signal of HIF-1α. Hypoxia can regulate apelin expression and this effect is closely correlated with the upregulation of HIF-1α [38–40]. HIF-1α increases the expression of the apelin gene [40]. Previous studies revealed that HIF-1α knockdown mitigates the expression of apelin and APJ to further depress hypoxia-induced BMSCs proliferation [17]. One recent study shows that hypoxia-induced upregulation of HIF-1α raises the expression of apelin and APJ, which serves as a key driver of EPCs proliferation to prevent hypoxic ischemic injury in vitro [41]. In this study, it was also discovered that there was a synchronizing increased expression of HIF-1α and apelin/APJ in the HP group in contrast with the normoxia group. Enhanced expression levels of HIF-1α, apelin, and APJ were observed after HP and during the cardiogenic differentiation process. SiRNA-mediated HIF-1α knockdown abolished HP-induced apelin and APJ expression. APJ was decreased in the condition of apelin knockdown. The above results corroborated that activation of the HIF-1α/apelin/APJ axis might execute an important role in HP-induced CSCs survival and cardiogenic differentiation.

## Conclusion

In summary, this study uncovered the influence of HP on CSCs biological activities in vitro. HP could facilitate CSCs survival and cardiogenic differentiation, and this effect could be partially attributed to activation of the HIF-1α/apelin/APJ axis. Further investigations into the role of the HIF-1α/apelin/APJ axis might be conducive for exploring novel strategies to improve CSCs transplantation efficiency.
